# Homolog of Pea *SGR* Controls Stay-Green in Faba Bean (*Vicia faba* L.)

**DOI:** 10.3390/genes14051030

**Published:** 2023-04-30

**Authors:** Jingbin Chen, Huimin Zhou, Xingxing Yuan, Yaming He, Qiang Yan, Yun Lin, Ranran Wu, Jinyang Liu, Chenchen Xue, Xin Chen

**Affiliations:** 1 Institute of Industrial Crops, Jiangsu Academy of Agricultural Sciences, Nanjing 210014, China; chenjingbin@jaas.ac.cn (J.C.);; 2 Jiangsu Key Laboratory for Horticultural Crop Genetic Improvement, Nanjing 210014, China; 3 College of Life Sciences, Nanjing Agricultural University, Nanjing 210095, China

**Keywords:** faba bean, stay-green, green cotyledon, *SGR*, chlorophyll degradation

## Abstract

Faba bean is an important legume crop consumed as a vegetable or snack food, and its green cotyledons could present an attractive color for consumers. A mutation in *SGR* causes stay-green in plants. In this study, *vfsgr* was identified from a green-cotyledon-mutant faba bean, SNB7, by homologous blast between the SGR of pea and the transcriptome of faba bean. Sequence analysis revealed that a SNP at position 513 of the CDS of *VfSGR* caused a pre-stop codon, resulting in a shorter protein in the green-cotyledon faba bean SNB7. A dCaps marker was developed according to the SNP that caused the pre-stop, and this marker was completely associated with the color of the cotyledon of faba bean. SNB7 stayed green during dark treatment, while the expression level of *VfSGR* increased during dark-induced senescence in the yellow-cotyledon faba bean HST. Transient expression of *VfSGR* in *Nicotiana. benthamiana* leaves resulted in chlorophyll degradation. These results indicate that *vfsgr* is the gene responsible for the stay-green of faba bean, and the dCaps marker developed in this study provides a molecular tool for the breeding of green-cotyledon faba beans.

## 1. Introduction

Faba bean (*Vicia faba* L.), also named broad bean, horse bean, or field bean, is an important cool-season legume crop that has a long history of cultivation [1]. At present, faba bean is the fourth most widely planted cool-season pulse in the world, and it is mainly grown in China, Ethiopia, and Australia [2]. Faba bean is rich in protein and energy [3,4]. Moreover, faba bean contains potentially therapeutic elements such as L-DOPA, which is the precursor of medicine for treating Parkinson’s disease [5]. Thus, faba bean is considered an important plant source of protein, which can help to fulfill the increasing worldwide demand for protein [6].

Faba bean has a huge genome which is up to 1C = 13.5 Pg (about 13 Gb) [7]. Recently, chromosome-scale assembly of the faba bean genome was released and revealed that up to 11.2 Gb of the genomic sequence was assembled in chromosomal pseudomolecules [8]. The large genome makes genetic and genomic studies of faba bean lag behind model plants. However, some studies on molecular marker development and the construction of genetic maps have been reported [9,10,11]. Due to the relatively limited information on the faba bean genome, very few faba bean genes have been reported so far. The gene *VC1* that controls vicine and convicine content in seeds was validated using genetic mapping and gene-to-metabolite correlations [12]. The seed coat tannin-related genes *zt-1*/*VfTTG1* and *zt-2*/*VfTTG8* were identified using comparative mapping and candidate gene approaches [9,13]. Moreover, *Vf_TFL1* was identified related to the terminal inflorescence of faba bean [14,15].

The green cotyledon of faba bean is controlled by a recessive gene, *il-1*, from a green faba bean in China [16]. The heredity of *il-1* is similar to that of *I*, which controls green cotyledons of pea (*Pisum sativum*) and was one of the traits analyzed by Gregor Mendel to develop the basic laws of genetic inheritance. The green cotyledon of pea was identified as a stay-green mutant with a lost function *sgr*, which was found co-segregating with the *I* locus [17,18]. Mutations in the homologs of *SGR*/*NYE1* causing stay-green have been found in many other plants, such as meadow fescue (*Festuca pratensis*) [17,19,20], *Arabidopsis* [21], rice (*Oryza sativa*) [22,23,24], tomato (*Solanum lycopersicum*) [25], pepper (*Capsicum annuum* L.) [26,27,28], *Medicago truncatula* [29], pea [18], soybean (*Glycine max*) [30], chickpea (*Cicer arietinum* L.) [31], common bean (*Phaseolus vulgaris* L.) [32], and Chinese cabbage/pakchoi (*Brassica campestris*) [33,34]. The PAO/phyllobilin pathway was found to be the major process involved in chlorophyll degradation [35]. In this pathway, SGR is a Mg-dechelatase, which is responsible for the first step of chlorophyll *a* (Chl*a*) degradation into pheophytin *a* by extracting magnesium (Mg) ions from Chl*a* [36]. SGR is also involved in the disassembly process of the light-harvesting chlorophyll-binding protein (LHCP) complexes during senescence [37]. Further research indicated that SGR was responsible for the degradation of photosystem I, while the degradation of the photosystem II core complex was independent of chlorophyll degradation mediated by SGR [38]. Chlorophylls in *sgr* mutants are retained in senescent green organs while their photosynthetic ability decreases. Thus, *sgr* is classified as a ‘type C’ stay-green mutant [39].

In this study, *VfSGR* was identified by blasting the pea *SGR* sequence against faba bean de novo transcript sequences reconstructed from RNA-seq data. A dCaps marker was developed according to the sequence variation of *VfSGR*. Additionally, the results of linkage analysis and association analysis indicated that *VfSGR* is a candidate gene that controls the stay-green of faba bean.

## 2. Materials and Methods

### 2.1. Faba Bean Materials and Phenotype Investigation

The green-cotyledon faba bean SNB7 was donated by Sun Yonghai from the Chuxiong Academy of Agricultural Sciences, Yunnan, China, via the material-sharing mechanism of China Agriculture Research System (CARS) —Food Legumes, and HST is a yellow cotyledon faba bean collected from Australia. An F_2_ population with 87 plants for genetic analysis was constructed from the cross between HST (♀) and SNB7 (♂). Germplasm of 191 faba bean accessions for association analysis of *VfSGR* was also collected via CARS—Food Legumes (Table S1).

The F_2_ population and germplasm of 191 faba bean accessions were sowed in 8 cm pods. Leaf samples for each of the plants were collected for DNA extraction after germination. Before sowing the seeds, a small part of the seed coat was removed and the color of cotyledons was recorded for each seed.

For dark-induced senescence, 45 day old plants of HST and SNB7 in 25 cm pods were transferred from normal-light conditions into a dark room (24 h dark, 20 °C). Six plants with a similar growing status for HST and SNB7 were selected for the experiment.

### 2.2. DNA Extraction, RNA Extraction, and cDNA Synthesis

The total DNA of faba bean was extracted using the CTAB method [40]. DNA samples were diluted to 5 ng/μL according to lambda DNA and analyzed using 1.0% agarose gel electrophoresis. Total RNA of faba bean was extracted using RNAprep pure Plant Kit (Tiangen, Beijing, China, DP432), following the product’s instructions. First-strand cDNA was synthesized using a Goldenstar^TM^ RT6 cDNA Synthesis Kit V.2 (Tsingke, Henan, China, TSK302S), following the kit’s instructions, and oligo (dT)_17_ was used as the primer.

### 2.3. Homology-Based Cloning of VfSGR

The sequence of the coding protein of pea SGR (NCBI GenBank accession no. BAF76351.1, named PsSGR here) was used as a query, and tBLASTn from BLAST+ (NCBI) was run against an assembled transcriptome of faba bean [10], using the default settings.

Polymerase chain reaction (PCR) was conducted to amplify the *VfSGR* using KOD-Plus-Neo DNA polymerase (Toyobo, Saitama, Japan, KOD-401), following the product’s instructions, and the annealing temperature was 58 °C. Primers were VfSGR_F1 (CCTCTCTTGTCTTCATTATTCATCA) and VfSGR_R1 (AGTCGCGTATAGACGAAGCAA). To amplify the mRNA sequence of *VfSGR*, cDNA was used as the template, and the extension time was 30 s. To amplify the genomic sequence of *VfSGR*, total DNA was used as the template, and the extension time was 2 min. PCR products were purified and Sanger sequenced by Tsingke Biotechnology Co., Ltd. (Zhengzhou, China).

Phylogenetic analysis was run using the ‘FastME/OneClick’ workflow of NGPhylogeny.fr (https://ngphylogeny.fr/, accessed on 27 April 2021) [41].

### 2.4. Real-Time PCR

Plants of faba bean for real-time PCR were planted in a growing chamber with a setting temperature of 20 °C and 14 h light/10 h dark light cycle. When the pods were almost mature (about 80 days after planting), roots, stems (3rd internodes from the top), old leaves (3rd leaves from the top), young leaves (newly expanded leaves), flowers, seeds (not mature), and pods (without seeds) were collected and stored in liquid nitrogen for RNA extraction.

Real-time PCR was run for verified expression levels of *VfSGR*. Two housekeeping genes, *Faba-actin* and *Gm-1-alpha*, were selected as internal references. The primers were CCCAGGAAATCTTCCTAGGACT and AGCCTTCAACACTACTGGCAA for *VfSGR*, GTTAGCAACTGGGATGACAT and GTTACGACCACTAGCATAGAGTG for *Faba-actin*, and GTGAAGCCCGGTATGCTTGT and CTTGAGATCCTTGACTGCAACATT for *Gm-1-alpha*. The reagent of real-time PCR was ChamQ Universal SYBR qPCR Master Mix (Vazyme, Nanjing, China, Q711-02), following the product’s instructions. Real-time PCR was performed using an ABI 7500 Real-Time System (Applied Biosystems, Foster City, CA, USA). Gene expression was analyzed using the 2^−ΔΔCT^ method [42]. Three biological repeats were detected for each sample.

### 2.5. dCaps Marker Design and Analysis

Primers of dCaps marker were designed with the help of dCAPs Finder 2.0 (http://helix.wustl.edu/dcaps/dcaps.html, accessed on 12 August 2020) [43]. The primers were dCaps_F: AAGTGTTTGATTTGATTTGATTAGG and dCaps_R: TACCCAAACTAATGACTCCTCTAATACCGG. A mismatched ‘A’ (underlined) was introduced into the dCaps_R primer to form an *Age*I recognition site (ACCGGT) in the PCR product of green-cotyledon faba beans. PCR was carried out using TSINGKE^®^ 2× Master Mix (Tsingke, Henan, China, TSE003). After that, 3 μL of PCR products was digested using endonuclease *Age*I-HF (NEB, Massachusetts, MA, USA, R3552). Those PCR products from green-cotyledon faba beans were recognized and digested by *Age*I-HF and produced shorter bands on 3% agarose gel.

### 2.6. Transient Expression

Plasmids for transient expression of VfSGR in *N. benthamiana* were constructed using the Gateway^®^ system. PCR was carried out using KOD Plus Neo (Toyobo, Japan, KOD-401). A common reverse primer (VfSGR-R: ggggaccactttgtacaagaaagctgggtTTACAAGTTACCATGTTGGG) was used for cloning the *VfSGR*. The recessive mutant *vfsgr^−SNB7^* was amplified using the primer VfSGR-SNB7-F (ggggacaagtttgtacaaaaaagcaggcttcATGGATACTCTAACAACCGCT) and VfSGR-R, while *VfSGR^−HST^* was amplified using the primer VfSGR-HST-F (ggggacaagtttgtacaaaaaagcaggcttcATGGATACTCTAACAACTGCTC) and VfSGR-R. Gateway^®^ BP recombination reaction was conducted to clone the PCR products into entry vector pDONR^™^221, and then the plasmids were transferred into *Escherichia coli*. After that, the pDONR^™^221 joint with target genes was extracted, digested, and linearized using endonuclease *Mlu*I-HF (NEB, Massachusetts, MA, USA, R3198). Subsequently, clones of *VfSGR* were connected to the transient expression vector pEarleyGate201 using Gateway^®^ LR reaction to construct the plasmids of 35S::*VfSGR^−HST^* and 35S::*vfsgr^−SNB7^*.

Transient transformation of 35S::*VfSGR^−HST^* and 35S::*vfsgr^−SNB7^* into leaves of *N. benthamiana* was conducted using *Agrobacterium rhizogenes* infiltration [37]. Empty pDONR^™^221 vector and infiltration buffer (mock) were used as negative controls. The infiltrated plants were cultivated in an incubator with a setting temperature of 25 °C and 16 h light/8 h dark light cycle. The third leaves from the top that were in a similar growing stage were selected for infiltration.

### 2.7. Measurement of Chlorophyll Content (SPAD)

The chlorophyll content of leaves was evaluated using SPAD-502 Plus (Konica Minolta, Osaka, Japan). For dark-induced senescence, a leaf with a similar growing stage from each plant was selected for SPAD evaluation. Selected leaves of six plants for each of HST and SNB7 were evaluated, and average values were calculated. For the *N. benthamiana* leaves of transient expression, five spots near infiltration sites were evaluated for SPAD values, and average values were calculated.

## 3. Results

### 3.1. Homology-Based Searching of VfSGR of Faba Bean

**Figure 1:**
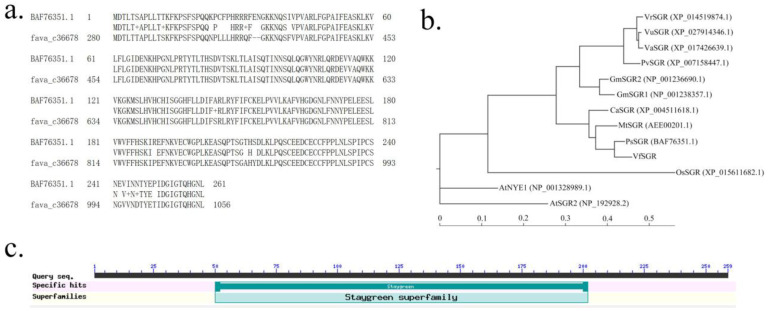
Homology-based searching of VfSGR of faba bean. (**a**) tBLASTn between PsSGR (BAF76351.1) of pea and transcript of VfSGR (fava_c36678) of faba bean. (**b**) Phylogenetic tree of SGRs of several plants. SGRs were from mungbean (*Vigna radiata*, Vr), cowpea (*Vigna unguiculata*, Vu), adzuki bean (*Vigna angularis*, Va), common bean (Pv), soybean (Gm), chickpea (Ca), *M. truncatula* (Mt), pea (Ps), faba bean (Vf), rice (Os), and *Arabidopsis thaliana* (At). NCBI accession numbers of the proteins are in brackets. (**c**) Conserved domain of faba bean VfSGR using CD-Search of NCBI.

The sequence of the coding protein of pea SGR (GenBank accession no. BAF76351.1, named PsSGR here) was used as the query, and tBLASTn was run against an assembled transcriptome of faba bean [10]. A transcript, *fava_c36678*, whose putative coding protein sequence showed 93% identity with PsSGR, was found to be the best hit (Figure 1a). Thus, here we named *fava_c36678* as *VfSGR*. While searching in the recently released reference genome sequence of ‘Hedin/2’ [8], *VfSGR* was relevant to the gene *Vfaba.Hedin2.R1.1g384200* located from 1,196,354,403 bp to 1,196,355,697 bp of chromosome Ch1L. The result of the phylogenetic analysis showed that the protein encoded by *VfSGR* was clustered with SGRs of several cold-season legumes, such as pea, *M. truncatula*, and chickpea (Figure 1b). The conserved domain of protein encoded by *fava_c36678* was analyzed using CD-Search (Conserved Domains Search) of NCBI [44] and a ‘Staygreen superfamily’ conserved domain was hit from residues No. 50 to No. 202 (Figure 1c). These results indicated that VfSGR is an SGR homolog in faba bean.

### 3.2. Expression and Sequence Polymorphism of VfSGR

**Figure 2:**
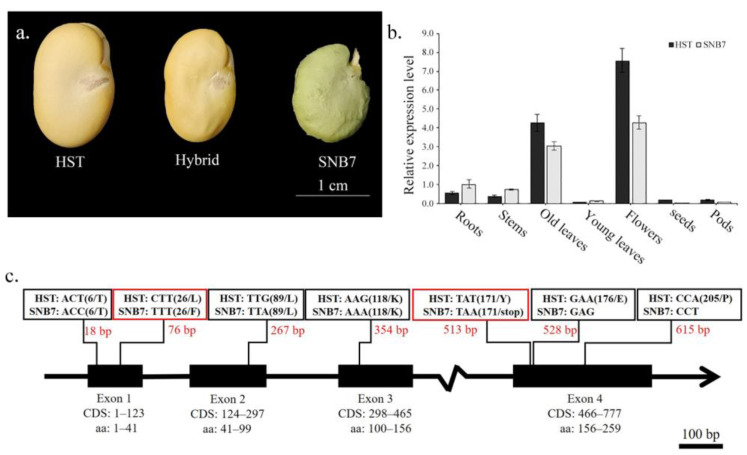
Sequence variation and expression pattern of *VfSGR*. (**a**) Color phenotypes of faba bean cotyledon. (**b**) Expression levels of *VfSGR* in different tissues of faba bean. (**c**) Variation in *VfSGR* DNA sequence between HST and SNB7. Black boxes indicate exons. Regions of CDS and amino acid residues (aa) are described under the exons. Numbers in red indicate the position of SNPs in CDS. For each SNP, relevant amino acid residues and their positions on the protein are shown in brackets. Red rectangles indicate the SNPs resulting in variations of the coding protein.

The expression pattern of *VfSGR* was analyzed by qPCR using different organs from yellow-cotyledon faba bean HST and green-cotyledon faba bean SNB7 (Figure 2a) as samples. Similar to the results of carnation (*Dianthus caryophyllus*), chrysanthemum (*Chrysanthemum morifolium*), and *Arabidopsis* [21,45,46], *VfSGR* was highly expressed in flowers (Figure 2b). *VfSGR* was more highly expressed in senescing old leaves than in young leaves of both HST and SNB7, suggesting that *VfSGR* was a senescence-related gene. However, the expression levels of *VfSGR* in roots, stems, young leaves, seeds, and pods were lower. Moreover, transcripts of *VfSGR* were more abundant in the old leaves and flowers of yellow-cotyledon HST than in those of green-cotyledon SNB7, suggesting that the premature stop codon in the mutant *vfsgr* could affect its expression.

Genomics and transcript sequences of *VfSGR* from yellow-cotyledon faba bean HST and green-cotyledon faba bean SNB7 were amplified and sequenced. By aligning the sequences between genomics sequences and transcript sequences, *VfSGR* of HST was found to consist of four exons and three introns, and coding a deduced protein containing 259 amino acid residues (aa) (Figure 2c, Supplementary File). While comparing coding sequences (CDS) of *VfSGR* between HST and SNB7, seven single nucleotide polymorphisms (SNPs) were detected (Figure 2c). Five of the SNPs were silent mutations that did not shift the protein coding, while the other two SNPs resulted in variations of the coding protein. For the green-cotyledon faba bean SNB7, the SNP on the position of 76 bp of CDS resulted in the coding shift from leucine (L) to phenylalanine (F) in the 26th aa. When aligning the coding protein sequences of *VfSGR* from 13 faba bean accessions (which were randomly selected from Table S1), the result showed that both of the two green-cotyledon faba beans (SNB7 and Touxinlü) were identical in the 26th aa (F), while the 11 yellow-cotyledon accessions were diverse between L and F. This result indicated that the SNP at the position of 76 bp was not associated with the color of the cotyledon (Figure S1). The SNP at 513 bp of CDS of *VfSGR* shifted the codon TAT that encodes tyrosine (Y) into a stop codon TAA, resulting in a shorter protein containing 170 amino acids in both the green-cotyledon faba bean SNB7 and Touxinlü (Figure 2c and Figure S1). This result indicated that the pre-stop codon was the reason for the green cotyledon.

### 3.3. Genetic Analysis of Vfsgr and Green Cotyledon of Faba Bean

A cross between yellow-cotyledon faba bean HST and green-cotyledon faba bean SNB7 was conducted, and the color of the cotyledon of hybrid F_1_ seeds was yellow (Figure 2a). In an F_2_ population of 87 plants, the cotyledons of 67 plants were yellow, while the cotyledons of 20 plants were green. The segregation ratio of yellow:green was 3:1 (*χ^2^* = 0.19, *P* = 0.66). These results demonstrated that the green color of cotyledons was controlled by a recessive gene, which was the same as a previous report [16].

**Figure 3:**
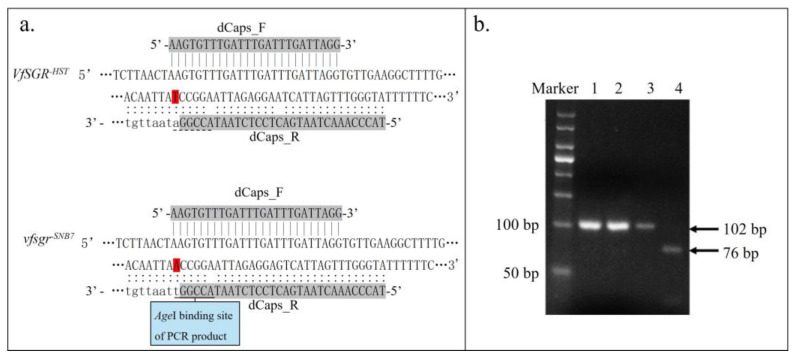
dCaps marker of *VfSGR*. (**a**) Diagram of the dCaps marker of *VfSGR*. Polymorphic nucleotides between *VfSGR^−HST^* and *vfsgr^−SNB7^* are marked in red. Lowercase letters indicate the nucleotides in PCR products. (**b**) Polymorphism of dCAPS marker of *VfSGR*. Lane 1, PCR product of HST before *Age*I digestion; Lane 2, PCR product of SNB7 before *Age*I digestion; Lane 3, PCR product of HST after *Age*I treatment; Lane 4, PCR product of SNB7 after *Age*I digestion.

A polymorphic dCaps marker was developed according to the SNP at position 513 bp of CDS of *VfSGR*, which resulted in the pre-stop codon (Figure 3a). PCR products were treated using endonuclease *Age*I, and a digested band of 76 bp from the green-cotyledon faba bean SNB7 was produced, while an undigested band of 102 bp emerged from the yellow-cotyledon faba bean HST (Figure 3b). The F_2_ population of 87 plants was detected individually using the dCaps marker, and the result showed that all 20 green-cotyledon plants generated digested SNB7 bands, while all 67 yellow-cotyledon plants generated undigested bands or heterozygous bands (Figure S2). Moreover, a natural population with 191 faba bean accessions (Table S1) was also analyzed using the dCaps marker. Among them, all 6 accessions with green cotyledons produced digested bands, while all 185 faba bean accessions with yellow cotyledons produced undigested bands (Figure S3). These results indicated that the SNP at 513 bp of ORF of *VfSGR*, which resulted in the pre-stop codon, was only detected in green-cotyledon faba beans, indicating that this SNP was associated with the colors of faba bean cotyledons.

### 3.4. The Involvement of VfSGR in Chlorophyll Degradation and Senescence

**Figure 4:**
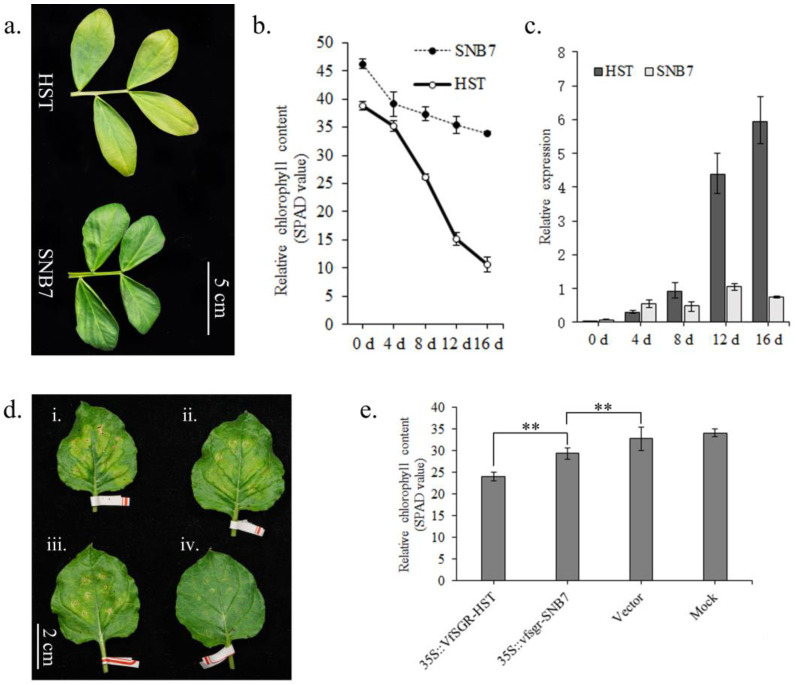
The involvement of *VfSGR* in chlorophyll degradation and senescence. (**a**) Leaves of faba bean after 16 days of dark-induced senescence. (**b**) Chlorophyll degradation throughout 16 days of dark treatment. (**c**) Relative expression levels of *VfSGR* throughout 16 days of dark treatment. (**d**) Transient expression of *VfSGR* in *N. benthamiana leaves*. i, ii, and iii are leaves infiltrated with *N. benthamiana* containing 35S::*VfSGR^−HST^*, 35S::*vfsgr^−SNB7^*, and empty vector pDONR™221, respectively; iv is a leaf infiltrated with the buffer (mock). (**e**) Chlorophyll content (SPAD) of *N. benthamiana* leaves after transient expression of *VfSGR*. LSD method for multiple comparisons, where ** denotes significant differences at the *p* < 0.01 level.

Dark-induced senescence has frequently been used as a model system to promote typical senescence symptoms such as chlorophyll degradation [47]. Throughout 16 d of dark treatment, leaves of SNB7 stayed green, while leaves of HST turned yellow after the 8th day of treatment (Figure 4a and Figure S4). Chlorophyll content was evaluated using the SPAD value, and the results showed that SPAD declined in both HST and SNB7 during the dark treatment. However, the SPAD value of HST declined faster than that of SNB7 (Figure 4b). These results indicate that the degradation of chlorophyll was more obvious in HST than in the stay-green mutant SNB7, which was similar to the results in *M. truncatula* [29].

To verify whether the expression level of *VfSGR* was related to the senescence of faba bean, qPCR was carried out for samples under different lengths of dark treatment (Figure 4c). The expression level of *VfSGR* was increased during the 16-day dark-induced senescence in both HST and SNB7. However, the expression levels of *VfSGR* were lower in the green-cotyledon SNB7 than in the yellow-cotyledon HST at almost all of the time points of the dark treatment, which indicates that the expression of *VfSGR* was depressed in the stay-green mutant SNB7 during the dark-induced senescence (Figure S4).

*VfSGR* was transiently expressed in *N. benthamiana* leaves. Six days after infiltration, yellowish phenotypes were observed in the third leaves from the top expressing *VfSGR* compared with mock samples treated with infiltration buffer and leaves infiltrated with empty vectors (Figure 4d). Moreover, *VfSGR^−HST^* functioned better than *vfsgr^−SNP7^*, as lower chlorophyll content (SPAD value) was detected in the leaves expressing *VfSGR^−HST^* (Figure 4e). These results indicated that *VfSGR* was involved in chlorophyll degradation and senescence. The mutation causing the pre-stop codon in *vfsgr^−SNB7^* weakened the function of chlorophyll degradation, which is the reason for stay-green of SNB7.

## 4. Discussion

In this research, the gene *vfsgr*, which is related to the green cotyledon of faba bean, was identified according to its homology with pea *sgr*, which is also responsible for the green cotyledon. *VfSGR* was expressed in different organs of faba bean, and it was more highly expressed in old leaves than in young leaves. Moreover, the expression of *VfSGR* was upregulated by dark-induced senescence in wild-type and *sgr* mutant leaves. These results were similar to the expression of *SGRs* in rice [24], *M. truncatula* [29], and pea [18], indicating that *VfSGR* is a senescence-related gene in faba bean.

Two SNPs shifting the coding of *VfSGR* were identified; one of them, at 513 bp of the ORF, which produced a stop codon ‘TAA’ and resulted in a shorter protein, was only detected in the sequence of green-cotyledon faba beans. This result indicated that the shorter protein was associated with the green cotyledon. In *Arabidopsis*, a conserved cysteine-rich motif (CRM, P-X3-C-X3-C-X-C2-F-P-X5-P) in the C-terminus of NYE1/SGR1 is indispensable for its function in chlorophyll degradation [48,49]. The mutation of each of the four cysteines in this motif affected the Mg-dechelating activity of NYE1/SGR1. In our study, truncation in the C-terminus of VfSGR caused the loss of the CRM motif (Figure S1). Similar to the SGRs of *Arabidopsis* and rice [37,49], transient expression of *VfSGR* of faba bean in *N. benthamiana* caused chlorophyll degradation, while the C-terminus-truncated vfsgr without CRM showed weaker function in chlorophyll degradation. These results indicate that the absence of CRM in the mutant vfsgr is the reason for stay-green in faba bean. However, transient expression of the C-terminus-truncated vfsgr also resulted in a slightly yellowish phenotype. It is possible that the truncated protein still had weak enzymatic activity, and abundant vfsgr protein triggered by strong promoter 35S caused the decline in chlorophyll content of the tobacco leaves.

Faba beans can be used as a vegetable or snack food, and their green cotyledon offers an attractive color for consumers. Moreover, stay-green pea plants were found to possess special proportions of nutritious substances [50,51], suggesting that faba bean with green cotyledons could be outstanding in terms of some nutrients. Therefore, the color of cotyledons could be one of the target traits of faba bean breeding. A thesis performing gene mapping revealed that the gene controlling the green cotyledon of faba bean was located between co-dominant markers CSSR6201 and SSR2833 [52]. While conducting BLAST of the recently released reference genome sequence of ‘Hedin/2′ [8] using primer sequences of the two markers as the queries, the relevant mapping region was found to be from 1.0 Gb to 1.3 Gb of chromosome Ch1L. The gene *VfSGR* that we studied was relevant to the gene *Vfaba.Hedin2.R1.1g384200* located at 1.2 Gb of Ch1L, and thus it was included in the mapping region. In our research, a dCaps marker developed on the SNP that produced pre-stop coding on *vfsgr* showed complete linkage to the color of cotyledons of faba bean in an F_2_ population. Moreover, this marker also showed a complete association with the color of cotyledons of faba bean in the natural population. However, all six accessions of the green-cotyledon faba bean we tested were collected from Yunnan Province, China (Table S1), which is the place there the green-cotyledon faba bean was first reported [16]. It is possible that these six accessions had the same origin and shared an identical allele of *vfsgr*. To further verify the association between *vfsgr* and the green cotyledon of faba bean, more alleles of *vrsgr* should be screened in the future. Our results of dCaps marker analysis further indicated that *VfSGR* was the key candidate gene that controls the color of cotyledons of faba bean. On the other hand, this dCaps marker could be used as an effective tool in selecting green cotyledon varieties of faba bean through back-cross breeding because the recessive mutant *vfsgr* in heterozygous yellow-cotyledon seeds could not be selected using visual observation. However, negative pleiotropic effects, such as compromised heat tolerance [53] and reduced germination rate [32,54,55], were also observed in the plants with lost-function *sgr*. Therefore, an approach for balancing the specific trait of stay-green and yield is still needed for the breeding program.

## Data Availability

All data are included either in the main text or as supplementary data. Other data can be requested from the corresponding author.

## Supplementary Materials

The following supporting information can be downloaded at: https://www.mdpi.com/article/10.3390/genes14051030/s1, Figure S1: Alignment of VfSGR protein sequences of faba bean with different cotyledon colors; Figure S2: dCaps marker analysis of F_2_ plants; Figure S3: dCaps marker analysis of 191 faba bean accessions; Figure S4: Dark-induced senescence of faba bean; Table S1: Faba beans for association analysis of *VfSGR*; Supplementary File: Genomic sequence and encoded protein of *VfSGR* of HST.
