# Supplementary material for: Homolog of Pea SGR Controls Stay-Green in Faba Bean (Vicia faba L.)
Source: Genes (Basel). 2023 Apr 30;14(5):1030. doi: 10.3390/genes14051030 (PMC10218623; doi:10.3390/genes14051030)
Supplement: Supplementary file 1 [file genes-14-01030-s001.zip › Supplementary_file.pdf]

Supplementary file Genomic sequence and encoded protein of *VfSGR* of HST

Genomic sequence of *VfSGR* of HST

Note: Sequences marked in yellow represent CDS domains of *VfSGR*. About 14 successive thymines (T) in the first intron could not be confirmed from the result of sequencing.

TGTGGTTTGATAATAAACACGTAACCTTGGTTTATTCTTTTGGTGTCCGCAA
AAATTGGATCTAGCTCAGGAACATGGATACTCTAACAACTGCTCCTTTGCTT
ACTTCTAAGTTCAAACCTTCGTTTTCTCCTCAACAAAATCCTCTTCTTCTAC
ATAGAAGACAGTTCGGGAAGAAGAATCAATCATTTGTTCCTGTATGTTTTTC
TG(~14T)GGATTTTTTAACAAAAAGTGTTTTGTGGATTTTAATGTTACTTTGT
ATTTGTTGGTTTTGTTTGGAAAAGGTTGCTAGGTTATTTGGACCAGCTATAT
TTGAAGCTTCAAAACTTAAGGTTTTGTTCTTAGGAATTGACGAAAACAAAC
ACCCAGGAAATCTTCCTAGGACTTATACGTTAACTCATAGTGATGTTACCTC
AAAACTCACTTTGGCAATTTCGCAAACCATAAACAACTCTCAGGTATTTCT
CTTGGTTTTCATTCTTATCAATATCATCATCATAAACCATATGAAAAGAACAA
CAAAAGAGGAACACAAAACAGGGTTGAAGAGGTGCACAATCACATCTTAG
TATTGATTTTTAGGGATACAAAAGTATGCTACAAACGTGTGAATTGGATTGC
AGTTGCAGGGATGGTACAATAGATTGCAAAGAGATGAAGTGGTTGCGCAAT
GGAAGAAGGTGAAGGGGAAAATGTCTCTGCATGTTCACTGTCACATTAGTG
GAGGCCATTTTCTTTTGGATATCTTTTCTAGACTTAGATACTTCATCTTCTGC
AAAGAGTTGCCAGTAGTAAGCATTTTCTTTTTTGAGTTTGTGGAATATTCTT
CTGAACTAGTATGTATTTGTATACTGTACTCTTAGTATATGATGTTTCTGTGTG
TCTGGTGTCCGTGTCTACTTAATACTCATGTAAGTATAGTGAGTTATAAAATA
AAATGTTTTGTTTCTGATTATCTGATCAAAATCTAAATGTTAATAGAGTGTTG
ATCTTAACTAAGTGTTTGATTTGATTTGATTAGGTGTTGAAGGCTTTTGTTCA
CGGTGACGGCAACTTATTCAACAATTATCCGGAATTAGAGGAATCATTAGTT
TGGGTATTTTTTCATTCAAAGATTCCAGAATTCAACAAGGTAGAATGTTGGG

GTCCACTAAAGGAGGCTTCACAACCAACTAGTGGGGCCCACTACGATTTGA

AACTACCACAATCTTGTGAAGAAGATTGTGAGTGCTGTTTTCCACCGTTGA

ATTTAAGCCCAATTCCGTGCTCTAATGGAGTTGTTAATGATACTTATGAAACT

ATTGATGGAATTGGAACCCAACATGGTAACTTGTAAACACACGATACTCTT

GTTGCTCGCATTAAACCCCGCCAACCTAAG

Deduced protein sequence of VfSGR of HST

MDTLTTAPLLTSKFKPSFSPQQNPLLLHRRQFGKKNQSFVPVARLFGPAIFEAS

KLKVLFLGIDENKHPGNLPRTYTLTHSDVTSKLTLAISQTINNSQLQGWYNRL

QRDEVVAQWKKVKGKMSLHVHCHISGGHFLLDIFSRLRYFIFCKELPVVLKA

FVHGDGNLFNNYPELEESLVWVFFHSKIPEFNKVECWGPLKEASQPTSGAHY

DLKLPQSCEEDCECCFPPLNLSPIPCSNGVVNDTYETIDGIGTQHGNL
