# Supplementary material for: Homolog of Pea SGR Controls Stay-Green in Faba Bean (Vicia faba L.)
Source: Genes (Basel). 2023 Apr 30;14(5):1030. doi: 10.3390/genes14051030 (PMC10218623; doi:10.3390/genes14051030)
Supplement: Supplementary file 1 [file Supplementary Figures.pdf › Supplementary Figures.pdf]

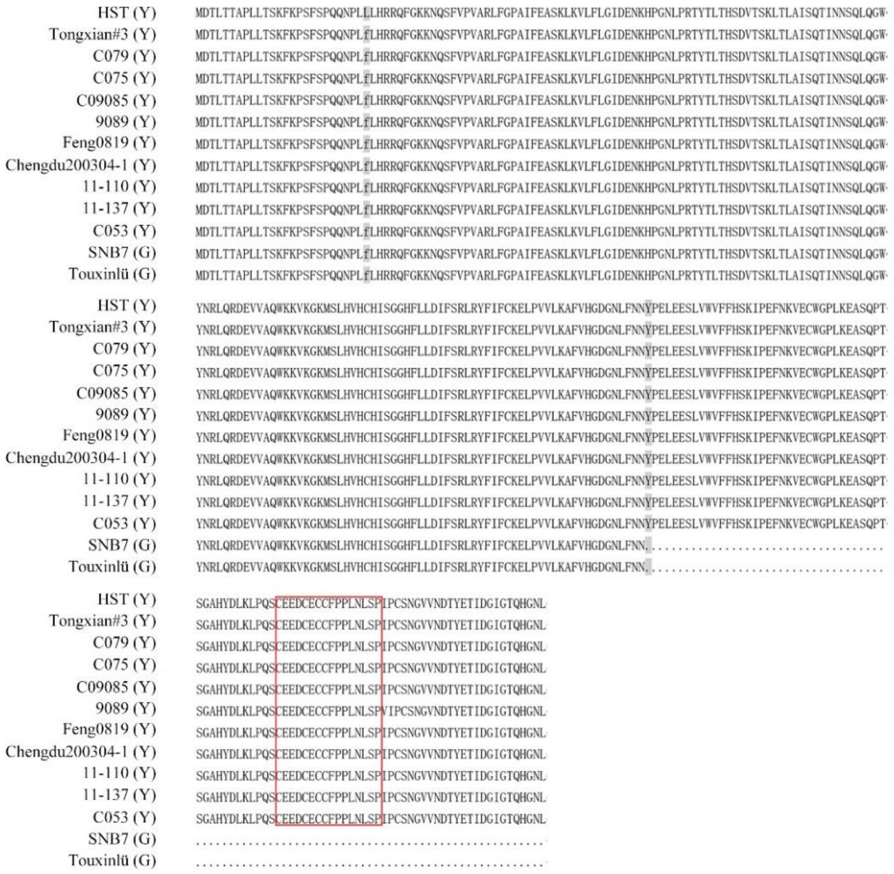

Figure S1 Alignment of VfSGR protein sequences of faba bean with different cotyledon colors. Y and G represent yellow cotyledon and green cotyledon, respectively. Red rectangle indicates the CRM motif of SGR [36,48]. Information of the 13 faba bean accessions is in Table S1.

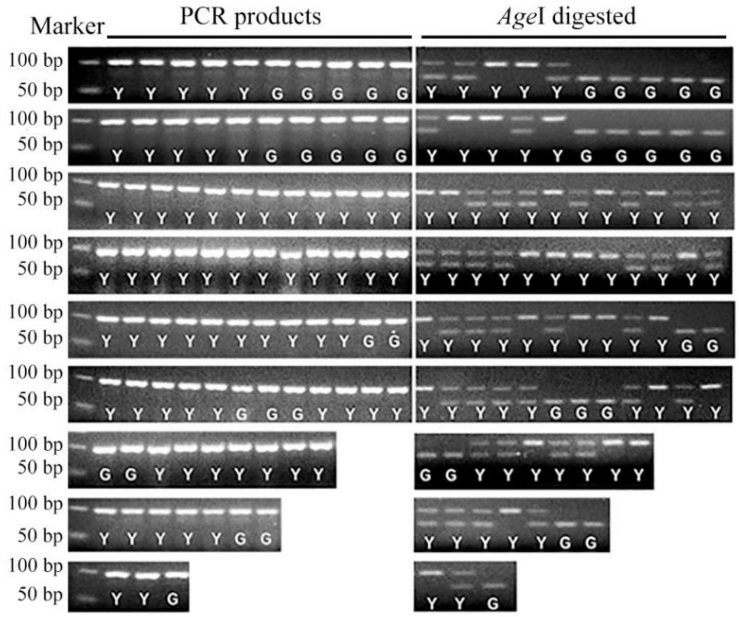

Figure S2 dCaps marker analysis of F_2 plants. Y and G indicate the plant with yellow cotyledon and green cotyledon, respectively.

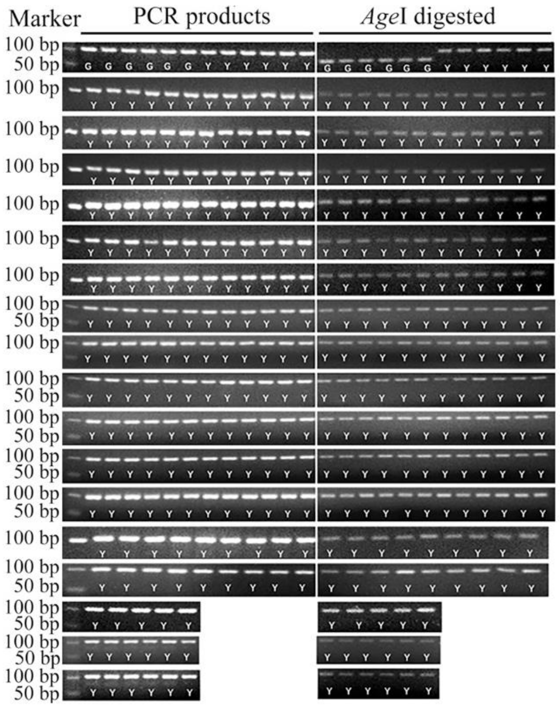

Figure S3 dCaps marker analysis of 191 faba bean accessions. Y and G indicate the plant with yellow cotyledon and green cotyledon, respectively. Accessions for analyzed are listed in Supplementary Table S1.

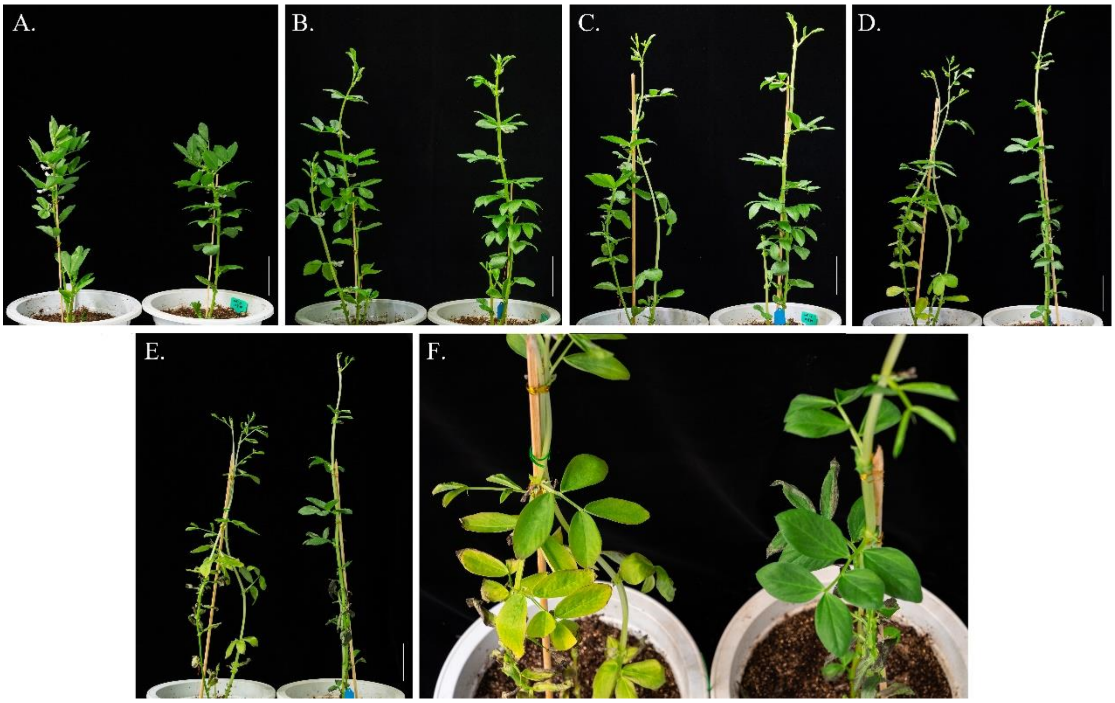

Figure S4 Dark-induced senescence of faba bean. A-E, p endured dark treatment days for 0 d, 4 d, 8 d, 12 d, and 16 d, respectively. F, plants endured dark treat ment for 16 d.
